# Supplementary material for: Unsupervised explainable AI for molecular evolutionary study of forty thousand SARS-CoV-2 genomes
Source: BMC Microbiol. 2022 Mar 10;22:73. doi: 10.1186/s12866-022-02484-3 (PMC8907386; doi:10.1186/s12866-022-02484-3)

**Fig. S1**. Results of PCA. Initial vectorial data for the BLSOM were defined as the first and second components from PCA, and the PCA results used for BLSOMs in Fig. 1 are presented. Nodes are colored as described in Fig. 1.


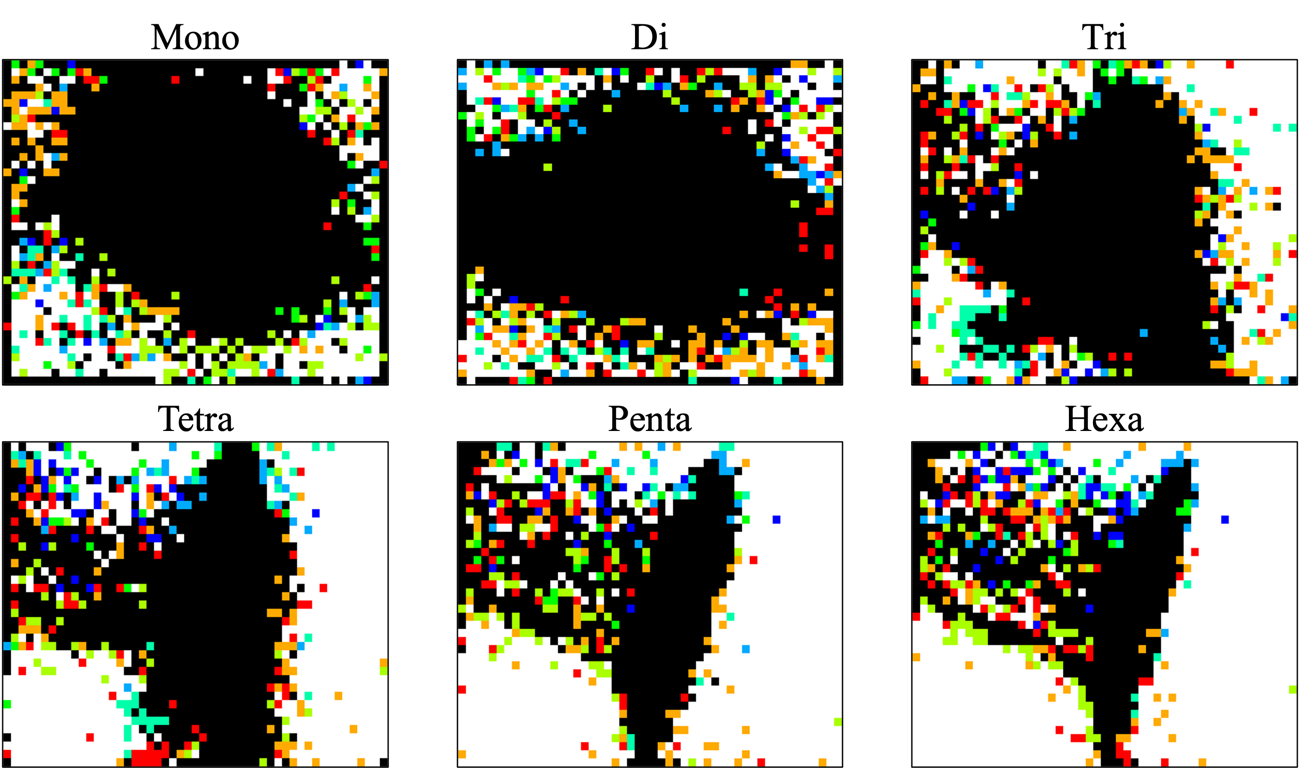


**Fig. S2**. Heatmaps of a portion of 7-mers for the BLSOM of 16,384 types of 7-mers presented in Fig. 2a. The choice of the “T” base was made for compatibility reasons with the NCBI reference genome notation, while the actual RNA base in the SARS-CoV-2 genome is a “U”. The representative vector for each node is composed of 16,384 (= 4^7^) variables, and contribution levels of the variables at each node are visualized by a heatmap as for Fig. 2aii: high (red), moderate (white) and low (blue).


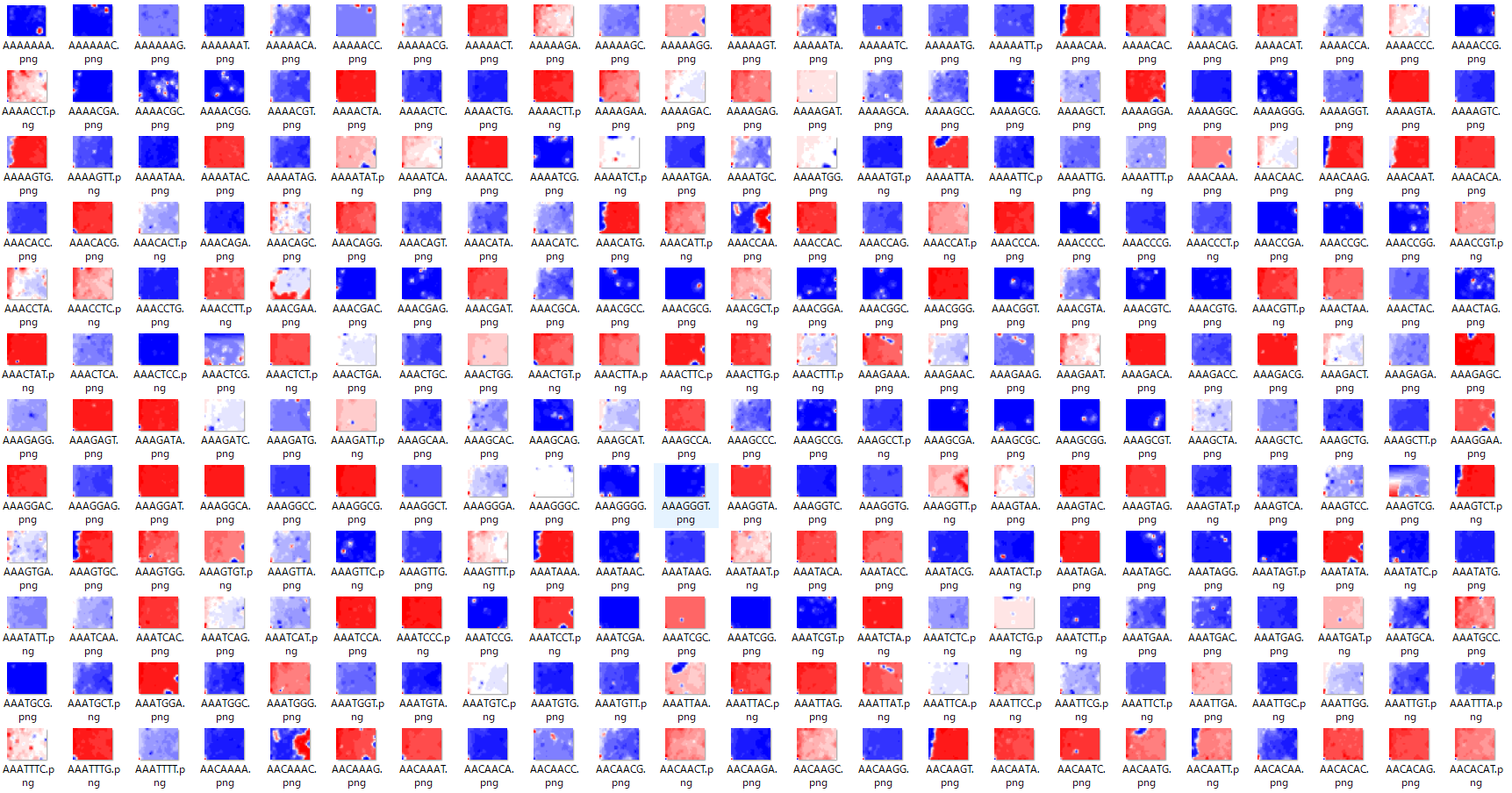


**Fig. S3.** Heatmaps of all 7-mers for the BLSOM of 377 different 7-mers presented in Fig. 2c.


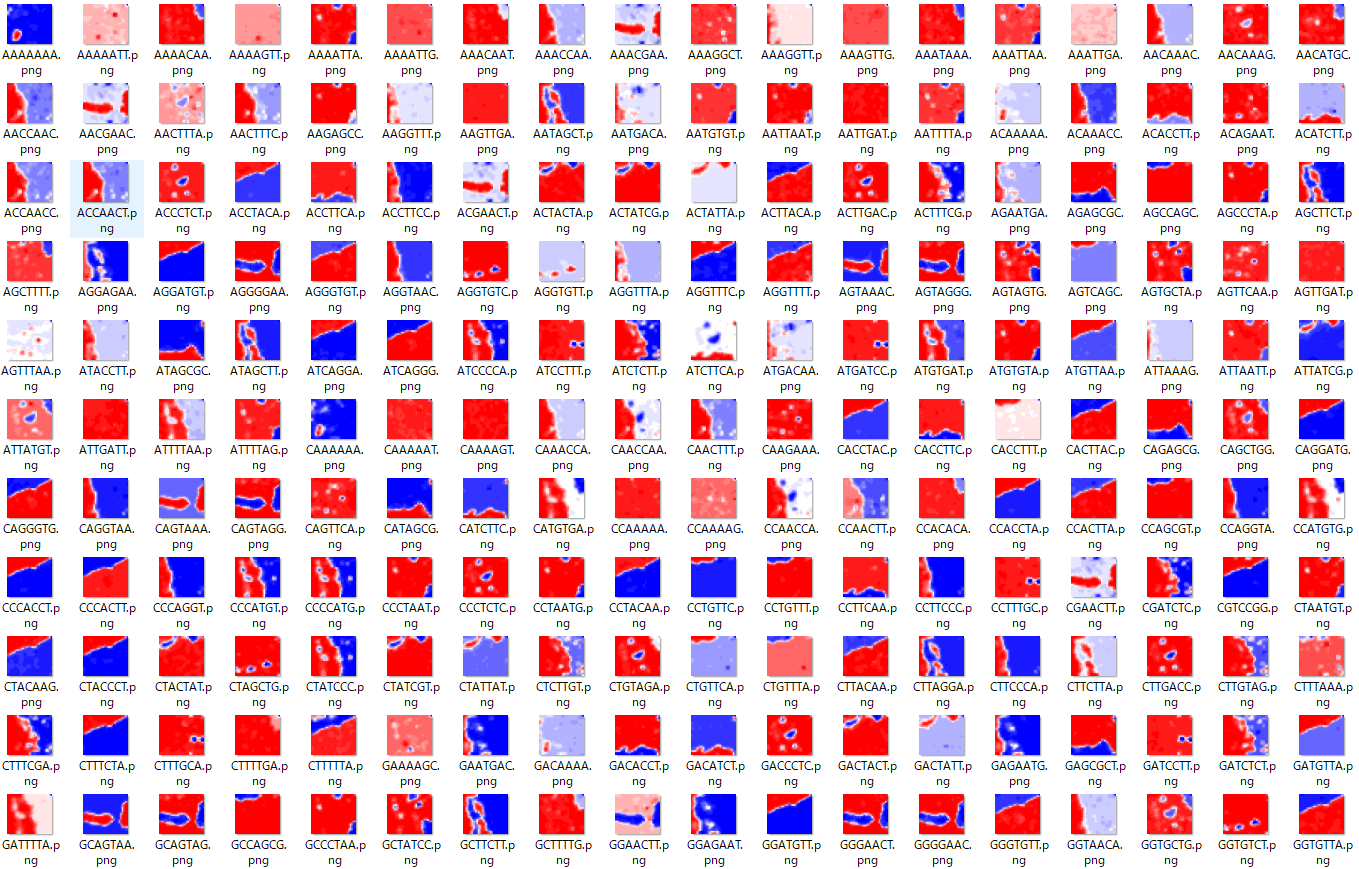

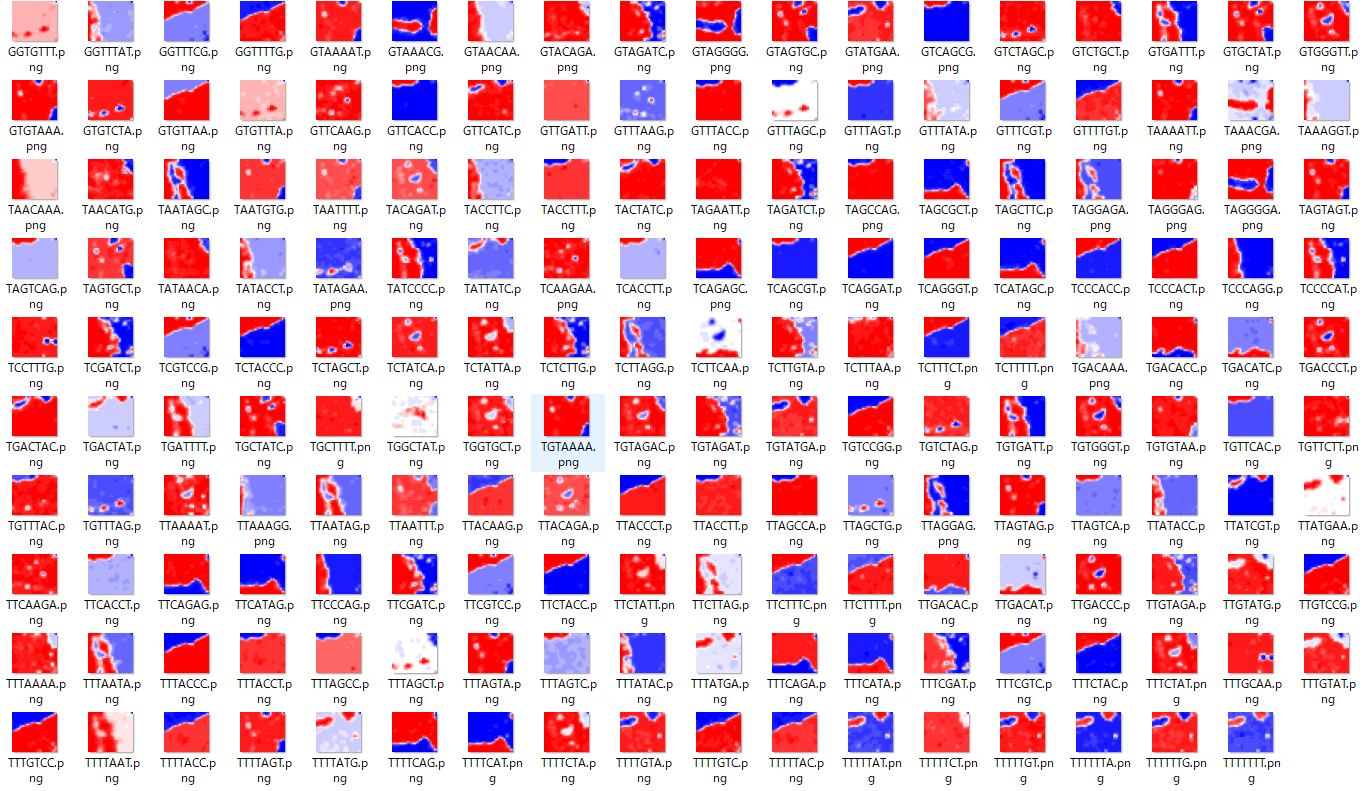


**Fig. S4.** BLSOM of 2087 types of 15-mers. The number of nodes was set as described in Fig. 2a. Nodes are colored as described in Fig. 1.


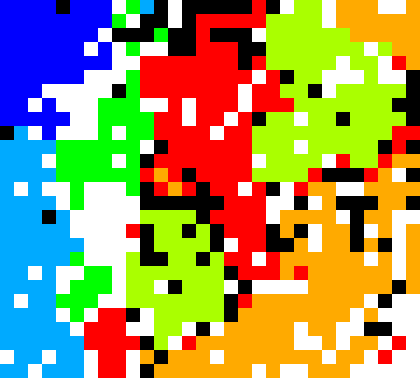

Supplement: Supplementary file 1 — Additional file 1: [file 12866_2022_2484_MOESM1_ESM.docx]
